# Bienestar emocional y social en la población indígena de Latinoamérica: una revisión sistemática

**DOI:** 10.1016/j.aprim.2026.103519

**Published:** 2026-05-19

**Authors:** Teseo Cardenas-Tambo, Martha L. Cervantes-Henríquez

**Affiliations:** a Universidad San Ignacio de Loyola, Lima, Perú; b Universidad Simón de Bolívar, Facultad de Ciencias Jurídicas y Sociales, Centro de Investigaciones en Ciencias de la Vida, Barranquilla, Colombia

**Keywords:** Salud indígena, Bienestar emocional y social, Seguridad cultural, Acción colectiva, Territorio, Latinoamérica, Indigenous health, Social and emotional wellbeing, Cultural safety, Collective action, Territory, Latin America

## Abstract

**Objetivo:**

Sintetizar la evidencia empírica sobre el bienestar emocional y social (SEWB) en las poblaciones indígenas de Latinoamérica y su vínculo con prácticas culturales, acciones colectivas y procesos comunitarios.

**Diseño:**

Revisión sistemática con síntesis narrativa, análisis temático y análisis bibliométrico; protocolo registrado en OSF (DOI: 10.17605/OSF.IO/UB7JD).

**Fuentes de datos:**

Scopus, Web of Science, PsycINFO, LILACS, SciELO y búsqueda manual en Google Scholar.

**Selección de estudios:**

Se incluyeron estudios empíricos en la población indígena latinoamericana publicados desde 2015. Se excluyeron estudios fuera de la región y documentos sin datos completos.

**Extracción de datos:**

Cribado en 2 fases (título / resumen y texto completo). Extracción de características y hallazgos. Riesgo de sesgo evaluado con MMAT.

**Resultados:**

Se identificaron 215 registros; se eliminaron duplicados (n = 19). Se evaluaron 23 textos completos y se incluyeron 19 estudios (Brasil = 7, Chile = 5, Colombia = 4, Ecuador = 1, México = 1, Perú = 1). Cabe destacar que la evidencia incluida se basó predominantemente en diseños de naturaleza cualitativa y transversal. Emergieron 3 ejes: territorio y espiritualidad como base del SEWB; identidad y memoria colectiva como factores protectores; y acción colectiva y organización comunitaria como mecanismos de cuidado.

**Conclusiones:**

El SEWB indígena se configura como un proceso relacional y situado con dimensiones territoriales, espirituales y comunitarias. Desde una perspectiva de atención primaria, los resultados sugieren que la expansión de la cobertura, sin pertinencia cultural y aceptabilidad, no garantiza acceso efectivo ni mejores resultados en salud. Persisten vacíos en longitudinalidad, medición culturalmente pertinente y evaluación de intervenciones, relevantes para orientar programas y servicios.

## Abstract (en)

**Objective:**

To synthesize empirical evidence on social and emotional wellbeing (SEWB) in Latin American Indigenous populations and its link to cultural practices, collective actions, and community processes.

**Design:**

Systematic review with narrative synthesis, thematic analysis, and bibliometric analysis; protocol registered in OSF (DOI: 10.17605/OSF.IO/UB7JD).

**Data sources:**

Scopus, Web of Science, PsycINFO, LILACS, SciELO, and manual searches in Google Scholar.

**Study selection:**

Empirical studies in Latin American Indigenous populations published since 2015 were included. Studies conducted outside the region and documents lacking complete data were excluded.

**Data extraction:**

Two-stage screening (title/abstract and full text). Extraction of characteristics and findings. Risk of bias was assessed using the MMAT.

**Results:**

A total of 215 records were identified; duplicates were removed (n = 19). Twenty-three full texts were assessed for eligibility, and 19 studies were included (Brazil = 7, Chile = 5, Colombia = 4, Ecuador = 1, Mexico = 1, Peru = 1). Notably, the included evidence was predominantly based on qualitative and cross-sectional designs. Three main themes emerged: territory and spirituality as the foundation of SEWB; identity and collective memory as protective factors; and collective action and community organization as mechanisms of care.

**Conclusions:**

Indigenous SEWB is configured as a relational and situated process with territorial, spiritual, and community dimensions. From a primary health care perspective, the results suggest that expanding coverage without cultural relevance and acceptability does not guarantee effective access or better health outcomes. Significant gaps remain in longitudinality, culturally relevant measurement, and intervention evaluation, which are relevant to guide programs and services.

## Introducción

Las poblaciones indígenas se definen como colectivos con continuidad histórica precolonial y vínculos territoriales profundos que ocupan una posición no dominante dentro del Estado^1^, ^2^. Mundialmente se estiman 476 millones de personas indígenas en aproximadamente 90 países, representando más de 5.000 comunidades y 4.000 lenguas^3^, ^4^. Sin embargo, estos grupos presentan históricamente una esperanza de vida de 5 a 15 años inferior a la de poblaciones no indígenas^5^, ^6^. Además, soportan una mayor carga de enfermedades crónicas y trastornos mentales^5^.

Ante este panorama, la atención primaria de salud (APS) constituye la estrategia fundamental para garantizar la efectividad, la equidad y la eficiencia de los sistemas sanitarios^7^, ^8^. Su funcionamiento óptimo depende de atributos esenciales conocidos como las 4 Cs: primer contacto, continuidad, coordinación e integralidad^9^. El cumplimiento de estos atributos se asocia directamente con mejores resultados de salud en la población general^10^. No obstante, al analizar la situación de los pueblos indígenas, se evidencia una brecha crítica en la equidad y el acceso efectivo^5^.

En el contexto específico de Sudamérica, la APS enfrenta barreras de aceptabilidad cultural que limitan su impacto^11^. Las altas tasas de suicidio, abuso de sustancias, conductas suicidas y depresión en la región sugieren que la competencia cultural tradicional ha sido insuficiente^12^, ^13^, ^14^, ^15^. Por ello, autores contemporáneos proponen transitar hacia la seguridad cultural, un enfoque que cuestiona las dinámicas de poder institucional^16^. Para lograr esta seguridad, es necesario incorporar el concepto de bienestar emocional y social (SEWB)^17^. Este constructo integra el equilibrio individual con la conexión comunitaria y territorial^18^.

Por lo tanto, el SEWB ofrece un marco prometedor, la evidencia empírica predominante sobre su aplicación proviene del Norte Global. Investigaciones en Australia y Nueva Zelanda demuestran que conectar con el territorio reduce la angustia psicológica^18^. Asimismo, estudios en Canadá y EE. UU. indican que las prácticas culturales actúan como factores protectores frente al trauma intergeneracional^19^.

Sin embargo, incluso en estos contextos, los programas a menudo priorizan habilidades individuales sobre la capacidad comunitaria^20^. Además, aunque se reconoce la necesidad de integrar perspectivas culturales en comorbilidades físicas, las intervenciones suelen ser fragmentarias^21^.

En Latinoamérica existen revisiones sistemáticas recientes sobre salud mental indígena, pero estas tienden a centrarse en la prevalencia epidemiológica de enfermedades o en el impacto de crisis sanitarias^12^. A diferencia de la literatura anglosajona, la región carece de una sistematización que utilice el marco del SEWB para analizar específicamente cómo la organización comunitaria actúa como determinante de salud. Este vacío es crítico, pues el contexto latinoamericano vincula el bienestar con la defensa territorial y la resistencia política^22^.

Esta visión dialoga con la psicología comunitaria, que entiende la salud como producto de la participación y el fortalecimiento colectivo^23^, ^24^. De igual forma, las epistemologías del Sur validan estos saberes locales frente a la hegemonía académica^25^.

Para abordar esta especificidad regional, este artículo emplea 2 nociones centrales. Primero, las acciones colectivas, entendidas como prácticas políticas mediante las cuales actores subalternos disputan significados y condiciones de vida^26^, ^27^. Segundo, los procesos comunitarios, definidos como dinámicas de participación y fortalecimiento que construyen agencia colectiva^23^, ^24^, ^28^.

El objetivo de esta revisión sistemática es mapear y analizar cómo estas prácticas, acciones y procesos contribuyen al bienestar emocional y social en comunidades indígenas latinoamericanas, con el fin de informar intervenciones que prioricen la autodeterminación y la justicia social.

## Metodología

Este artículo siguió la guía PRISMA 2020^29^ y PRISMA-P 2015 para asegurar transparencia y reproducibilidad desde el diseño del protocolo^30^. El protocolo se registró en OSF para control de versiones y acceso abierto^31^, reduciendo duplicación de esfuerzos según recomendaciones Cochrane^32^.

Las búsquedas se ejecutaron en Scopus, Web of Science, PsycINFO, LILACS, SciELO y Google Scholar empleando la ecuación: (TITLE-ABS-KEY(«emotional social well being» OR «emotional well-being» OR «social well-being») AND TITLE-ABS-KEY((indigenous OR «indigenous communities» OR «native population» OR «First Nations» OR «tribal communities» OR «comunidades indígenas» OR «nativos americanos»))).

### Criterios de inclusión

Estudios empíricos (cualitativos, cuantitativos, mixtos) o literatura gris con rigor metodológico; centrados en comunidades indígenas autoidentificadas de Latinoamérica (comprendiendo geográficamente a los países de América del Sur, América Central y México, independientemente de su edad). que describan prácticas, acciones colectivas o procesos relacionados con bienestar emocional y social; publicados después de enero 2015.

### Criterios de exclusión

Estudios fuera de Latinoamérica; que no aborden bienestar emocional / social o sus componentes (estudios puramente epidemiológicos sin factores protectores o exclusivamente farmacológicos); editoriales, cartas, comentarios o resúmenes sin datos completos.

La exclusión de la literatura puramente epidemiológica obedeció a una delimitación conceptual intencionada, estructurada para no diluir el enfoque del estudio y centrar el análisis en los determinantes psicosociales, comunitarios y de resistencia, más que en la prevalencia clínica.

La selección se realizó en 2 fases (título / resumen y texto completo) por 2 revisores independientes, resolviendo discrepancias por consenso. Aunque no se calculó formalmente un coeficiente estadístico de concordancia interevaluador, dado que se adoptó un enfoque basado en el diálogo directo, esta decisión se reconoce como una limitación metodológica menor. Se empleó Rayyan para gestión y cribado^33^, Excel para trazabilidad, Zotero para organización bibliográfica y Mixed Methods Appraisal Tool (MMAT) para evaluar riesgo de sesgo^34^. El protocolo incluyó un análisis bibliométrico con VOSviewer; no obstante, debido al número limitado de estudios y la heterogeneidad de los metadatos, el mapa de coocurrencia no fue realizado por falta de robustez^35^, ^36^.

## Síntesis y análisis de datos

Para la síntesis de la evidencia, se llevó a cabo un análisis temático cualitativo. En una primera fase, se realizó una lectura a profundidad de los resultados de los estudios incluidos para extraer y generar códigos iniciales (etiquetas) vinculados a las prácticas, acciones colectivas y determinantes del bienestar. Posteriormente, mediante un proceso iterativo, estos códigos iniciales se compararon y agruparon progresivamente para conformar categorías analíticas más amplias. Todo el proceso de organización, refinamiento de códigos y la consolidación de los 3 ejes temáticos finales se apoyó operativamente en matrices de Microsoft Excel®, garantizando la trazabilidad de los datos sin el empleo de un software de análisis cualitativo especializado.

## Resultados

**Figura 1:**
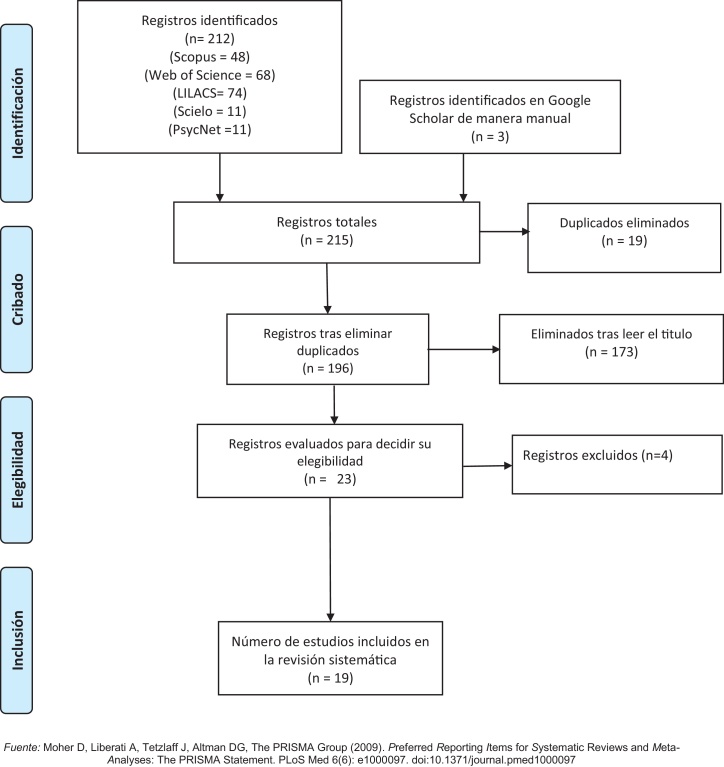
Diagrama de flujo Prisma.

El proceso de selección de estudios se presenta en la figura 1. En la fase de elegibilidad se evaluaron 23 registros a texto completo, excluyéndose 4: un editorial, sin datos empíricos sobre salud mental en la población latinoamericana; 2 revisiones (teórica y de alcance), sin datos primarios sobre pueblos indígenas; y un estudio empírico realizado fuera de Latinoamérica. Por consiguiente, 19 estudios cumplieron los criterios de inclusión y fueron incorporados en la revisión sistemática. De manera global, el cuerpo de evidencia sintetizado se caracterizó por un predominio estructural de estudios con enfoques cualitativos y diseños transversales.

La calidad metodológica y el riesgo de sesgo de los estudios incluidos se evaluaron mediante la MMAT, versión 2018^34^. Los resultados de la evaluación por criterios (C1-C5), según el diseño de cada estudio, se presentan en la tabla 1. Dado que la síntesis de resultados integra evidencia proveniente de estudios con calidades metodológicas heterogéneas (bajas, medias y altas), sus hallazgos fueron interpretados de manera conjunta; se reconoce que esto podría limitar el peso inferencial de ciertas conclusiones.

**Tabla 1:** Evaluación de la calidad metodológica de estudios

Estudio	Diseño (Categoría MMAT)	C1	C2	C3	C4	C5	Calidad general
37	Cualitativo (Pesquisa / ação)	Sí	Sí	Sí	Sí	Sí	Alta
38	Cualitativo (Hermenéutico)	Sí	Sí	Sí	Sí	Sí	Alta
39	Cualitativo (CBPR / decolonial)	Sí	Sí	Sí	Sí	Sí	Alta
40	Cualitativo (IAP)	Sí	Sí	No	No	No	Baja
41	Cualitativo (Estudio de caso)	Sí	Sí	Sí	Sí	Sí	Alta
42	Cualitativo (Cartografía)	Sí	Sí	Sí	Sí	Sí	Alta
43	Cualitativo (Inv. intervención)	Sí	Sí	Sí	Sí	Sí	Alta
44	Cuantitativo (No aleatorizado)	Sí	Sí	No	No	Sí	Media
45	Cuantitativo (Correlacional)	Sí	Sí	Sí	No se sabe	Sí	Alta
46	Cuantitativo (Descriptivo)	Sí	Sí	Sí	Sí	Sí	Alta
47	Cualitativo (Etnografía)	Sí	Sí	Sí	Sí	Sí	Alta
48	Cuantitativo (Descriptivo)	No	No	No	No se sabe	No	Baja
49	Cuantitativo (Ecológico)	Sí	Sí	Sí	Sí	Sí	Alta
50	Cualitativo (Etnográfico)	Sí	Sí	No	No	No	Baja
51	Cualitativo (Etnográfico)	Sí	Sí	Sí	Sí	Sí	Alta
52	Cuantitativo (Descriptivo)	Sí	Sí	Sí	Sí	Sí	Alta
53	Cualitativo (Decolonial)	Sí	Sí	Sí	Sí	Sí	Alta
54	Cualitativo (Grupos focales)	Sí	Sí	Sí	Sí	Sí	Alta
55	Cualitativo (Etnografía)	Sí	Sí	Sí	Sí	Sí	Alta

C1-C5 refieren a los criterios específicos de cada diseño según la herramienta MMAT.

MMAT: Mixed Methods Appraisal Tool.

A continuación, se describen las acciones colectivas, procesos comunitarios y resultados principales de los estudios incluidos, los cuales se detallan en la tabla 2.

**Tabla 2:** Acciones colectivas, procesos comunitarios y determinantes del bienestar en los pueblos indígenas de Latinoamérica

Referencia	País	Acciones colectivas	Procesos comunitarios	Resultados
49	Brasil (Ceará)	Uso de la red de atención psicosocial (RAPS); Equipos de salud indígena.	Cobertura de servicios de atención primaria; Identificación de indicadores epidemiológicos.	Alta prevalencia de suicidio y violencia pese a mayor cobertura, indicando necesidad de adecuación cultural.
37	Brasil (Ceará)	Realización de «Rodas de conversa» y rituales del Toré; Reivindicación política por tierras.	Fortalecimiento de vínculos comunitarios vía ancestralidad; Conexión espiritual con el territorio.	La salud mental indígena es colectiva y territorial. La falta de territorio genera sufrimiento ético / político.
38	Chile (Biobío)	Participación en asociaciones indígenas urbanas; Ceremonias colectivas (nguillatunes); Juego de palin.	Resignificación de identidad individual / colectiva; Conexión simbólica ancestral; Solidaridad frente al Estado.	El bienestar subjetivo se liga a la valoración positiva de la identidad Mapuche. Imaginario de «malestar» estructural.
39	Chile	Koyang (parlamentos); Nietun (revitalización); Marichiweu (resistencia).	Newen (fuerza espiritual); Azmapu (ley ancestral); Reciprocidad y defensa de la dignidad.	La resiliencia comunitaria es resistencia y transformación social ante el racismo. El bienestar se basa en la justicia social.
40	Colombia (Sucre)	Talleres de historia, música y danza Zenú; Círculos de conversación.	Fortalecimiento del sentido de pertenencia; Valorización de tradiciones; Integración social escolar.	Aumento de autoestima y orgullo cultural. Identidad cultural fuerte como pilar del bienestar psicológico.
41	Brasil (Bahía)	Grupos colaborativos ante desastres; Capacitación para agentes de salud.	Construcción participativa de cuidados; Integración de cosmologías (espiritualidad, territorialidad) en emergencias.	Guías prácticas (EPREP) para proteger el «Buen Vivir» en desastres, validando el protagonismo comunitario.
42	Brasil (Bahía)	Lucha por la tierra (retomadas); Organización para autodefensa; Rituales del Toré.	Espiritualidad (encantados); Autonomía política; Cohesión social frente a la violencia externa.	La lucha por la tierra produce salud mental. La organización comunitaria mitiga traumas («resistencia es salud»).
43	Brasil (Bahía)	Acciones de vigilancia territorial; Movilización de líderes para gestión de salud.	Fortalecimiento de la identidad étnica en conflicto; Cuidado colectivo; Protagonismo en salud.	La salud mental es inseparable de la lucha territorial. El «Buen Vivir» implica autonomía y rechazo a la servidumbre.
44	México (Yucatán)	Diseño colaborativo de intervención; Difusión de salud mental vía WhatsApp®.	Adaptación cultural (maya) de contenidos; Reducción del estigma social; Apoyo social.	Reducción significativa de depresión y ansiedad. Validación de intervenciones digitales culturalmente adaptadas.
45	Chile (Araucanía)	Participación en el movimiento social Mapuche; Apoyo a protestas.	Identidad colectiva politizada; Percepción de discriminación como factor de unión.	Una fuerte identidad colectiva actúa como factor protector para el bienestar psicológico frente a la discriminación.
53	Brasil (Rio G. do Norte)	Trabajo colectivo (mutirões); Fútbol femenino como socialización.	Reciprocidad; Relacionalidad con la tierra; Cuidado comunitario ampliado.	«Bien Vivir» es «ver al otro bien». La salud mental emerge de los vínculos comunitarios y el territorio.
46	Chile (Araucanía)	Estudio cuantitativo sobre conductas alimentarias.	Relación entre comportamientos alimentarios (atracones) y factores psicológicos.	Prevalencia de atracones menor en Mapuches. Mapuches con atracones reportan peor bienestar emocional.
47	Perú (Amazonía)	Organización estudiantil (OEPIAP); Protestas y tomas de rectorado.	Capital social mediante organización estudiantil; Apoyo mutuo entre pares; Resistencia a la discriminación.	El bienestar requiere recursos y buenas relaciones. La organización es clave para sobrevivir al choque cultural.
48	Ecuador (Saraguro)	Rituales de sanación con aguacoya; Ceremonias de limpia y florecimiento.	Conexión con la cosmovisión andina; Catarsis emocional colectiva; Reafirmación de identidad.	Los rituales generan paz y claridad mental. La salud mental es equilibrio cósmico y social.
50	Colombia (La Guajira)	Resolución de conflictos (Pütchipü’üi); Actividades económicas tradicionales.	Organización matrilineal (clanes); Transmisión oral de costumbres; Solidaridad colectiva.	Bienestar social deficiente por falta de servicios, sostenido por la cohesión cultural y la identidad étnica.
51	Colombia (Nariño)	Diálogos de saberes (mingas); Grupos focales con taitas y mamas.	Interculturalidad; Armonización de medicina ancestral y psicología; Recuperación de la «tulpa».	La salud mental requiere equilibrio espiritual. Modelo donde psicología y saberes ancestrales dialoguen sin jerarquías.
54	Colombia (Sierra Nevada)	Grupos focales participativos; Iniciativa «Renacer Kankuamo».	Ley de Origen; Armonía naturaleza / humanos; Autodeterminación política y sanitaria.	El «Buen Vivir» es paz y armonía con el entorno. Lo material es secundario frente a la salud espiritual / territorial.
55	Brasil (Mato Grosso)	Sistema de grupos de edad; Ritos de paso; Caza colectiva.	Tradicionalismo como base del bienestar; Relaciones de mentoría; «Sufrir juntos» para crear lazos.	El bienestar social incluye esfuerzo y lucha. La salud individual a veces se sacrifica por el bienestar social.
52	Chile	Compromiso cultural, pedagogías interculturales y participación comunitaria centrada en el buen vivir.	Satisfacción microsistémica, efectos de aculturación y discriminación, y equilibrio vital entre comunidad y naturaleza.	Mayor bienestar no indígena, diferencias culturales significativas, variaciones por género y disminución etaria.

La producción científica analizada presenta una distribución geográfica concentrada principalmente en Latinoamérica, con Brasil liderando el número de publicaciones (n = 6), seguido por Chile (n = 5) y Colombia (n = 4). En un segundo nivel se ubican EE. UU. (n = 3), Ecuador y España (ambos con n = 2). Finalmente, Canadá, México, Países Bajos, Perú y Reino Unido registran una publicación cada uno (n = 1). Cabe señalar que el recuento se basa en la afiliación institucional de los autores. Posteriormente, se realizó un análisis detallado de los componentes «Teoría», «Contexto», «Características» y «Metodología», el cual se presenta en la tabla 3.

**Tabla 3:** Matriz TCCM

Componente	Descripción detallada y analítica
Teoría (T)	Los artículos emplean una variedad de enfoques teóricos, agrupados en 3 grandes corrientes, por ejemplo, el principio del «Buen Vivir» (Sumak Kawsay / Suma Qamaña) como pilar ético / político y de salud mental 49, 53, 54, 51, 41 y los Estudios Decoloniales para analizar las estructuras de poder y conocimiento 53.
	Se aplican marcos como el Enfoque de Capacidades de Amartya Sen para evaluar libertades y funcionamientos 54, el modelo de bienestar psicológico de Ryff 40, la teoría del desarrollo psicosocial de Erikson 48, y la antropología del bienestar 55.
	Se utiliza la psicología comunitaria para entender las dinámicas colectivas 37, 51, la teoría de la identidad social para explorar la pertenencia y la discriminación 38, 45, y la praxis crítica de resiliencia comunitaria (CCRP) para analizar respuestas transformadoras a la adversidad 39.
Contexto (C)	Los estudios se distribuyen en 6 países, con un fuerte enfoque en Brasil y Chile. Los contextos específicos son: Brasil: Nordeste (Ceará, Bahía, Rio Grande do Norte), en comunidades rurales e indígenas (Pataxó, Tupinambá, Potiguara, Tapuya Kariri), a menudo en situaciones de post-desastre (inundaciones en 41) o conflicto por la tierra 43. En comunidades mapuche (Chile), tanto en contextos urbanos (Gran Concepción) como rurales, abordando temas de racismo estructural y desastres 39, 45.
	Colombia: En resguardos indígenas (Kankuamo, Zenú, Guachucal) y comunidades Wayuu, explorando la vida cotidiana, la cultura y los servicios de salud 54, 51, 40, 50.
	Ecuador, México y Perú: Estudios focalizados en comunidades específicas como Saraguro (rituales de sanación y equilibrio cosmo-social), Maya (intervención digital culturalmente adaptada durante la pandemia) y Awajún (organización estudiantil indígena en contextos universitarios) 48, 44, 47.
Características (C)	Se analizan indicadores epidemiológicos como suicidio e intoxicación 49, depresión y ansiedad 44, sufrimiento psíquico 37 y el impacto de la discriminación 45.
	Conceptos y prácticas de bienestar: El eje central son las comprensiones ancestrales del bienestar 37 y los imaginarios sociales 38. Se exploran la resiliencia cultural 39, los rituales 48, la participación comunitaria 51, la relacionalidad y reciprocidad 53 y la espiritualidad 43.
	Se abordan el conflicto por tierras 43, las líneas de cuidado en salud pública 41, las trayectorias educativas 47 y el rol del liderazgo y las redes de apoyo 50.
	Factores estructurales y políticos: 45, la autoestima y pertenencia 40, la satisfacción vital 52 y la autoeficacia 46.
Metodología (M)	Se observa una diversidad metodológica, con predominio de ciertos enfoques: Enfoques cualitativos (predominantes): Etnografía 51, 55, estudio de caso 41, cartografía social 43 y análisis de contenido / documental 37. Se utilizan entrevistas y grupos focales como técnicas principales.
	Enfoques cuantitativos: Uso de encuestas y escalas psicométricas para medir variables como bienestar subjetivo, discriminación y salud mental, con análisis de regresión y correlacionales^45^, ^46^, ^49^, ^52^.
	Enfoques participativos y mixtos: Se emplean la investigación / acción^37^, ^40^, la co-creación y el diálogo de saberes^54^, reflejando un compromiso con la producción de conocimiento colaborativo.
	Las muestras varían desde estudios de caso con pocos participantes (N= 6)^53^ hasta encuestas a gran escala (N > 44,000)^52^.

El análisis TCCM identifica vacíos que limitan la interpretación y aplicabilidad de la evidencia sobre bienestar emocional y social indígena. En «Teoría», predomina el anclaje en enfoques decoloniales y «Buen Vivir», pero la articulación con modelos psicológicos operativos y la explicación de mecanismos psicosociales permanece limitada^48^, ^50^. En «Contexto», la evidencia se concentra en Brasil, Chile y Colombia, con subrepresentación de países con alta población indígena y escasa atención a escenarios urbanos y migración interna, lo que reduce la transferibilidad regional^38^, ^42^. En «Características», se priorizan descripciones de factores asociados sin capturar procesos dinámicos como la conflictividad territorial, y persisten tensiones de medición al emplear instrumentos estandarizados que no siempre reflejan los marcos indígenas de bienestar^49^, ^52^. En «Metodología», predomina el diseño cualitativo y transversal, con limitada evaluación de intervenciones y escasez de diseños longitudinales, lo que restringe la capacidad de orientar políticas basadas en evidencia, pese a algunas experiencias aplicadas^44^. A partir de estos vacíos, se propone la siguiente agenda de investigación (tabla 4).

**Tabla 4:** Agenda de investigación

Componente	Dirección de investigación	Justificación
Teoría (T)	Integrar marcos decoloniales, «Buen Vivir» y bienestar emocional y social con modelos psicológicos de bienestar, formulando un marco híbrido culturalmente pertinente para pueblos indígenas latinoamericanos.	La evidencia sintetizada describe el bienestar como relacional, territorial y espiritual; la falta de integración teórica con modelos psicológicos limita la operacionalización del constructo sin reducirlo a enfoques individualistas^37^, ^48^, ^53^, ^54^.
	Desarrollar teorías interculturales multinivel que expliquen mecanismos mediante los cuales acciones colectivas y procesos comunitarios influyen en bienestar emocional y social bajo condiciones estructurales locales.	La revisión identifica acciones colectivas y procesos comunitarios, pero persiste un vacío en modelos explicativos de mecanismos y condiciones de contexto, lo que dificulta traducir hallazgos a intervenciones en servicios^48^, ^50^.
Contexto (C)	Ampliar la investigación a países subrepresentados en la evidencia disponible, priorizando territorios amazónicos, andinos y de frontera social en Perú, Bolivia y Paraguay, con descripción contextual detallada.	La concentración de estudios en Brasil, Chile y Colombia limita la diversidad regional y reduce la transferibilidad del conocimiento a otros sistemas y territorios latinoamericanos^38^, ^42^.
	Analizar migración interna y transiciones rural / urbano en las poblaciones indígenas latinoamericanas y su asociación con bienestar emocional y social, considerando cambios de redes comunitarias y acceso a servicios.	La falta de estudios sobre movilidad interna y urbanización dificulta comprender cómo se transforman identidad, soporte comunitario y acceso efectivo al cuidado en escenarios frecuentes para atención primaria^38^, ^47^.
Características (Ch)	Implementar diseños longitudinales que evalúen trayectorias de bienestar emocional y social en relación con espiritualidad, identidad cultural, conflicto territorial y discriminación, con seguimiento temporal suficiente.	La evidencia predominante es transversal o cualitativa; la escasez de longitudinalidad limita inferencias temporales y el diseño de intervenciones sostenibles^37^, ^41^, ^52^.
	Desarrollar y validar indicadores cuantitativos culturalmente pertinentes de bienestar emocional y social para pueblos indígenas latinoamericanos, con equivalencia intercultural y utilidad para evaluación de programas.	La ausencia de medición estandarizada adaptada culturalmente dificulta comparar contextos, evaluar intervenciones y monitorear impacto sin distorsionar el constructo^49^, ^52^.
Metodología (M)	Diseñar y evaluar intervenciones culturalmente pertinentes con enfoque pragmático en escenarios reales, incorporando participación comunitaria y criterios de factibilidad para su aplicación en atención primaria.	La escasez de estudios de intervención y la heterogeneidad metodológica limitan la generación de evidencia aplicable; diseños pragmáticos favorecen aplicabilidad y escalamiento^44^.
	Aplicar métodos mixtos con integración explícita de datos cualitativos y cuantitativos, incluyendo triangulación y reporte de integración, en estudios multicéntricos latinoamericanos.	La dependencia de diseños cualitativos y la validación limitada de instrumentos reducen robustez y generalización; métodos mixtos integrados fortalecen la evidencia sin sacrificar pertinencia cultural^51^, ^54^.

## Discusión

Esta revisión sugiere que la defensa del territorio y la espiritualidad operan como ejes explicativos centrales del bienestar emocional indígena, al potenciar la agencia colectiva y la identidad cultural^37^, ^49^, ^54^. Los hallazgos indican que la salud mental en estos contextos trasciende la ausencia de enfermedad para configurarse como un proceso relacional de resistencia política y equilibrio comunitario, donde la autonomía y la memoria ancestral actúan como factores protectores frente a la inequidad sistémica^55^^,56^.

Aunque la revisión no restringió la evidencia a escenarios asistenciales, los resultados tienen implicancias directas para la APS, dado su rol como primer nivel de contacto y como eje de la puerta de entrada, la continuidad y la coordinación del cuidado^7^. Al analizar la literatura bajo los atributos esenciales de la APS (las 4 Cs), emergen conexiones críticas^9^.

Se sugiere que la integralidad del servicio se expanda más allá de lo estrictamente clínico hacia una dimensión ecológico-social, en la medida en que el bienestar se concibe como un equilibrio dinámico con el territorio y con entidades espirituales vinculadas a la cosmovisión indígena^37^, ^54^. Asimismo, la continuidad del vínculo terapéutico se sostiene en el reconocimiento de la memoria cultural como un soporte central del cuidado, más que en la mera adherencia a dispositivos biomédicos estandarizados^53^. En este marco, la coordinación efectiva de las intervenciones requiere orientarse hacia una articulación activa con las redes comunitarias y los sistemas propios de atención, dado que el bienestar no se produce a nivel exclusivamente individual, sino que emerge como una construcción colectiva anclada en relaciones sociales, políticas y territoriales^38^, ^47^.

Esta visión holística, documentada en contextos diversos, desde los Andes (Saraguro) hasta la Amazonía (Tupinambá) y el Caribe (Wayuu) redefine la salud mental como un proceso situado^39^, ^43^, ^50^, ^55^. La sanación en estas comunidades privilegia el espíritu y la energía vital, desafiando la fragmentación biomédica^22^. Para el atributo de primer contacto en APS, esto implica que la accesibilidad no es solo geográfica, sino cultural.

Sin embargo, se ilustra una paradoja crítica para la gestión sanitaria: municipios con alta cobertura de servicios de salud aún presentan elevadas tasas de suicidio indígena^49^, ^52^. Este hallazgo es central para la APS, pues sugiere que la expansión de la cobertura, sin aceptabilidad ni adecuación, es insuficiente para garantizar resultados efectivos^10^. La disponibilidad de infraestructura no equivale a acceso efectivo si el servicio carece de pertinencia.

En consecuencia, el reto transita de la competencia cultural hacia la seguridad cultural. Este marco es necesario para abordar las barreras identificadas en los estudios, como la discriminación, la distancia lingüística y la desconfianza derivada de la violencia estatal^11^, ^16^, ^38^. Una APS culturalmente segura debe reconocer que la lucha territorial observada en los casos Potiguara y Tupinambá^42^, ^43^, ^53^, no es un evento externo, sino un determinante de la salud mental que requiere validación clínica y apoyo psicosocial, no patologización.

En cuanto a las implicancias prácticas, la identidad cultural se perfila como un factor protector robusto. La evidencia señala que la revitalización de la lengua y la memoria histórica fortalece la resiliencia y amortigua sintomatología depresiva^39^, ^45^. En ausencia de evidencia longitudinal suficiente, se plantean implicancias potenciales para la APS: el fomento de espacios de encuentro comunitario y el co-diseño de intervenciones que incorporen saberes ancestrales como recursos terapéuticos válidos^51^. Experiencias como el Sistema Indígena de Salud Propio Intercultural en Colombia ilustran la viabilidad de esta convergencia^50^.

No obstante, estas interpretaciones deben considerarse con cautela, debido a que son escasos los estudios con diseños longitudinales que evalúen el impacto causal de estas articulaciones. El componente metodológico muestra un predominio de enfoques cualitativos y transversales^37^, ^41^ frente a un número limitado de intervenciones evaluadas^45^, ^46^, ^49^. El análisis de riesgo de sesgo revela heterogeneidad; mientras los estudios cualitativos aportan validez interna sobre la experiencia subjetiva, otros presentan debilidades en el control de variables^48^. Esta variabilidad indica que persiste el desafío de sistematizar estos hallazgos con el rigor necesario para su generalización en políticas públicas. Se reconoce una limitación derivada de la estrategia de búsqueda empleada: al privilegiar términos explícitos asociados a la nomenclatura *well-being* o SEWB, es posible que se haya excluido literatura académica latinoamericana relevante que conceptualiza el bienestar desde marcos ontológicos locales (como buen vivir, equilibrio espiritual o salud comunitaria) sin utilizar dichas etiquetas estandarizadas.

## Conclusiones

Esta revisión sintetiza evidencia que conceptualiza el bienestar emocional y social indígena como un fenómeno relacional y situado, estrechamente vinculado al territorio, la espiritualidad, la identidad cultural y los procesos comunitarios. En los estudios incluidos, estos elementos aparecen como recursos centrales para la resiliencia y la agencia colectiva frente a condiciones estructurales adversas, más que como atributos exclusivamente individuales.

Para la toma de decisiones en salud, los hallazgos sugieren que la traducción del conocimiento a programas y servicios requiere evitar reduccionismos biomédicos y priorizar enfoques culturalmente pertinentes, con participación comunitaria y articulación con redes locales. Sin embargo, la evidencia disponible es heterogénea y mayoritariamente cualitativa y transversal, con escasas evaluaciones de intervención. A estos vacíos se suma la limitación conceptual de la estrategia de búsqueda, que pudo dejar fuera constructos locales al priorizar el término SEWB, y el hecho de haber analizado estudios de calidad metodológica variable de forma paralela. Se recomienda fortalecer la agenda regional con diseños longitudinales, medición culturalmente pertinente y evaluaciones aplicadas que permitan estimar impacto y transferibilidad a políticas y servicios.

## Financiación

Esta investigación no recibió financiamiento específico de agencias públicas, comerciales ni sin fines de lucro.

## Contribuciones de autor (CRediT)

Teseo Cardenas-Tambo: conceptualización; metodología; investigación; curación de datos; análisis formal; visualización; redacción del borrador original.

Martha L. Cervantes-Henríquez: conceptualización; metodología; validación; supervisión; redacción, revisión y edición; administración del proyecto.

## Consentimiento informado

No fue necesario obtener consentimiento informado, dado que el estudio no involucró participantes humanos ni datos identificables.

## Consideraciones éticas

Este estudio corresponde a una revisión sistemática de la literatura científica publicada. No se recolectaron datos primarios ni se involucró la participación directa de seres humanos, por lo que no fue necesaria la aprobación por parte de un comité de ética.

## Uso de inteligencia artificial

Se utilizó inteligencia artificial generativa únicamente como apoyo en la mejora de la redacción, claridad y estilo del manuscrito. No se empleó para la generación de contenido científico, análisis de datos, interpretación de resultados ni toma de decisiones académicas. Los autores asumen plena responsabilidad sobre el contenido del artículo.

## Conflicto de intereses

Los autores declaran no tener conflictos de intereses.
